# Beneficial Effects of Olive Oil and the Mediterranean Diet on Alzheimer’s Disease and Vascular Dementia: A Review

**DOI:** 10.3390/medicina62040696

**Published:** 2026-04-04

**Authors:** Aitor González-Cidad, Juan Carlos García-Moncó, Gustavo C. Román

**Affiliations:** 1 Faculty of Medicine and Nursing, University of the Basque Country UPV/EHU, 48940 Leioa, Spain; agonzalez615@ikasle.ehu.eus; 2 Department of Neurology, Basurto University Hospital, 48013 Bilbao, Spain; hospit05@sarenet.es; 3 Department of Neurology, Houston Methodist Neurological Institute, Houston, TX 77030, USA

**Keywords:** aging, cognitive decline, dementia, mild cognitive impairment, neuroprotection, polyphenols, stroke, vascular risk factors

## Abstract

*Background and Objectives:* During the past 25 years, a significant body of research has been conducted reporting on the salutary effects of the Mediterranean diet and extra-virgin olive oil, one of its main components. The initial studies were epidemiological observations on populations with very low mortality rates due to significant reductions in myocardial infarction fatalities. Population-based studies demonstrated that the Mediterranean diet with olive oil consumption is associated with a lower prevalence of cardiovascular and cerebrovascular disease, obesity, arthritis, and cancer. *Materials and Methods:* In this narrative review, we present recent studies on the effects of extra-virgin olive oil and the Mediterranean diet—compared with various other diets—on several vascular risk factors, including hypertension, hyperlipidemia, type 2 diabetes mellitus, and obesity, as well as their impact on cognitive decline and dementia. *Results:* This diet has been shown to improve cognitive function in patients with mild cognitive impairment, Alzheimer’s disease, vascular cognitive impairment, and vascular dementia. The main mechanisms responsible for cognitive improvement include control of arterial hypertension by reducing systolic and diastolic blood pressure, lowering triglycerides and low-density lipoprotein cholesterol and increasing high-density lipoprotein cholesterol, along with improvement in fasting glucose, insulin levels, and hemoglobin A1c in subjects with type 2 diabetes mellitus, as well as lowering body mass index and obesity. *Conclusions:* The Mediterranean diet and olive oil induce—along with prevention of cardiovascular disease and stroke—a significant improvement of vascular risk factors, slowing the progression of both vascular dementia and Alzheimer’s disease. There is a need for additional placebo-controlled clinical trials to confirm the supportive nutritional role of extra-virgin olive oil in age-associated cognitive decline in the elderly.

## 1. Introduction

In 2019, we published two comprehensive reviews [1,2] of a large number of observational epidemiological studies demonstrating the overall salutary effects of the Mediterranean diet (MedDiet) and extra-virgin olive oil (EVOO) consumption on the decrease in overall mortality rates and coronary heart disease fatalities, as well as on lowering the incidence rates of cardiovascular and cerebrovascular diseases, cancer prevalence, arthritis and age-associated cognitive decline.

Recent reviews on dementia and mild cognitive impairment (MCI) by Jones et al. [3] and on vascular dementia (VaD) by Ng et al. [4] have identified a common set of modifiable risk factors for dementia. The most common one includes vascular risk factors, which are central to the pathogenesis of VaD but also contribute significantly to AD and other dementias. These modifiable risk factors include arterial hypertension [5]—the major risk factor at the population level—as well as dyslipidemia and high body mass index (BMI), all of which can trigger inflammation and stenosis of cerebral blood vessels, leading to reduced blood flow and neural tissue damage. A prior history of stroke is regarded as the strongest vascular risk factor for developing VaD and AD [6]; nevertheless, metabolic disorders such as diabetes mellitus also contribute significantly by inducing systemic damage to the cerebral microvasculature. In addition to vascular risks, obstructive sleep apnea and psychological disturbances—such as depression—induce chronic stress and inflammation, while lifestyle factors including smoking and alcohol abuse cause direct injury to the cerebral vasculature and brain structures [3,4]. By targeting these shared vascular and metabolic pathways, lifestyle interventions offer a powerful strategy for the prevention and management of the most significant risk factors for dementia.

Despite these advancements, significant gaps remain in the recent literature; while the general benefits of the MedDiet are well-documented, there is a need to synthesize the most recent data (2020–2026) regarding the specific neuroprotective mechanisms of EVOO and how these interventions correlate with measurable clinical outcomes. This review distinguishes itself from prior work by synthesizing the significant volume of recent evidence. We integrate a broad amount of evidence, ranging from the latest meta-analyses involving over 730,000 participants [7] to newly studied MRI evidence [8], demonstrating how the MedDiet influences the shared vascular and neurodegenerative risk factors. This review explores the role of the MedDiet and EVOO in mitigating these risk factors and the pathophysiological mechanisms involved, along with their potential to serve as pharmaceutical and dietary interventions.

## 2. Materials and Methods

For this updated narrative review, we employed a comprehensive search strategy across major databases, including PubMed, Google Scholar, EMBASE, Cochrane, Web of Science Core Collection, and Scopus. The literature search concluded on 20 February 2026 and was restricted to full-text articles published in English or Spanish. The search was conducted using a combination of terms, including “Alzheimer’s disease”, “vascular dementia”, “mild cognitive impairment”, “metabolic syndrome”, “extra-virgin olive oil”, “polyphenols”, and “Mediterranean diet adherence”. The primary objective was to evaluate the effects of the MedDiet and its core components—specifically EVOO—on MCI, AD, vascular cognitive impairment (VCI), and VaD. To ensure the rigor of this narrative review, we applied the following inclusion and exclusion criteria:

Inclusion Criteria: We included peer-reviewed randomized clinical trials (RCTs), observational studies, systematic reviews, and meta-analyses. Studies were required to focus on human populations (general, elderly, or high-risk groups such as those with T2DM or MetS) and investigate the impact of the MedDiet or EVOO on cardiovascular risk factors, metabolic outcomes, or cognitive performance. Preclinical studies (murine models) were included to provide mechanistic explanations for neuroprotective effects.

Exclusion Criteria: We excluded study protocols, case reports, editorials, and articles without full-text access. Studies published before 2020 were excluded for sections regarding cardiovascular and metabolic risk factors to maintain currency, except for landmark trials (e.g., PREDIMED) or studies with direct cognitive and stroke outcomes, where no strict date limit was applied.

Study Selection and Quality Assessment: Two reviewers independently screened titles and abstracts, followed by a full-text review of relevant articles. Evidence from high-quality sources was prioritized, emphasizing meta-analyses with large participant pools (e.g., >700,000 participants) and landmark randomized controlled trials to minimize bias. Terminology was standardized according to the VasCog-2-WSO criteria [9] to ensure conceptual consistency across selected studies. The outcomes synthesized from these sources include cardiovascular and metabolic risk factors (hypertension, dyslipidemia, obesity, and stroke), psychological disturbances (depression and sleep disorders), and cognitive outcomes measured via standardized assessments such as the Mini-Mental State Examination (MMSE).

### Terminology

This review adopts the terminology recently established by the VasCog-2-WSO criteria [9]. Cognitive decline refers to the general reduction in cognitive performance from a previous baseline. Under the term Vascular Contributions to Cognitive Impairment and Dementia (VCID), we distinguish between Preclinical/At-Risk VCID, Mild Cognitive Impairment, and VaD. In contrast, VaD (or Major VCI) is reserved for severe cognitive impairment of vascular origin that significantly disrupts instrumental activities of daily living (typically ≥2 SDs below norms). AD is a distinct process characterized by amyloid and tau pathology, though it frequently coexists with vascular changes. Such events are shown to precipitate AD’s pathogenesis in the form of mixed dementia [10], which is a primary focus of this review.

## 3. Results: Mediterranean Diet (MedDiet)

### 3.1. MedDiet Effects on the Main Risk Factors for Dementia

#### 3.1.1. Cardiovascular Risk Factors (CVRFs)

The influence of the MedDiet on most of the CVRFs is multifactorial, as demonstrated by major randomized controlled clinical trials (RCTs) such as PREDIMED [11] (PREvención con DIeta MEDiterránea) and CORDIOPREV [12] (CORonary Diet Intervention with Olive Oil and Cardiovascular PREVention). These large RCTs have consistently reported a lower incidence of cardiovascular events, myocardial infarction and stroke, along with a reduction in overall mortality and an increase in life expectancy. The protective role of the MedDiet and EVOO against these vascular events is best substantiated by PREDIMED [11], a landmark RCT involving nearly 7500 individuals at high cardiovascular risk. This study demonstrated a 30% relative risk reduction in major adverse cardiovascular events among participants adhering to the MedDiet supplemented with EVOO or nuts, compared to the control group. Similarly, the CORDIOPREV study [12] further confirmed these results with the composite hazard ratio (HR = 0.72) showing a greater protective effect of the MedDiet over a low-fat diet in secondary prevention, particularly highlighting long-term benefits on inflammatory markers and lipid profiles.

The MedDiet has been shown to induce significant reductions in weight, BMI, and waist circumference [13,14,15,16,17]. These changes are fundamental, given that obesity and central adiposity [18,19] are well-established risk factors for cardiovascular disease, also causing an increase in the frequency of obstructive sleep apnea, another major CVRF. The MedDiet was shown by a meta-analysis of RCTs [16] to be effective in reducing BMI and obesity among children and adolescents, significantly lowering the relative risk (ARR = 0.12). These effects are not limited to the younger population, given that higher MedDiet adherence was correlated with a 9% lower risk of developing obesity over 5 years in adults [20]. Furthermore, the MedDiet profoundly impacts blood pressure, with several studies showing significant reductions in blood pressure, measured both in-office (−15.1 mmHg) and ambulatory, resulting in decreases in both systolic (−3.1 mmHg) and diastolic (−1.6 mmHg) values when compared to the usual diet [21,22]. Retrospective case–control studies [23] also showed evidence that lower adherence to the MedDiet was present in hypertensive patients, with data showing a mean adherence score of 3.8, compared to 6.9 in the non-hypertensive group. Among those with hypertension, the decreased compliance with the MedDiet also correlated with higher rates of dyslipidemia, chronic kidney disease (CKD), and other vascular diseases.

Improvement in lipid profiles is another positive effect of adherence to the MedDiet. The results include reductions in triglycerides and low-density lipoprotein (LDL) cholesterol, along with an increase in beneficial high-density lipoprotein (HDL) cholesterol, as demonstrated by several meta-analyses [24,25]. The effects on the lipid profile were modest and seem to be potentiated by calorie restriction and physical activity. The MedDiet-only group had minor decreases in small-dense low-density lipoprotein cholesterol (sd-LDL-C = 0.13 mmol/L) at 12 months, while the MedDiet group engaged in a low-calorie diet and increased physical activity achieved greater reductions in sd-LDL-C (0.21 mmol/L) at 6 months [26]. Other objective ways to measure the positive effects of the MedDiet are emerging, such as those used in a recent prospective cohort study derived from the PREDIMED trial [27], demonstrating that an increase in polyphenol-derived metabolites in urine was inversely associated with the incidence of CVRFs.

#### 3.1.2. Anti-Inflammatory and Antioxidant Effects

The Composite Dietary Antioxidant Index (DAI)—a strong predictor of antioxidant capacity—is found to be elevated in the MedDiet due to its rich content of vitamins A, C, E, carotenoids, zinc, and selenium [28]. The DAI is inversely associated with the major vascular risk factors, including hyperlipidemia, obesity, T2DM and hypertension [28,29]. Furthermore, a higher DAI is linked to a lower risk of stroke and a reduction in Intima-Media Thickness of the Common Carotid (IMT-CC), particularly in individuals with higher BMI [30,31].

The anti-inflammatory mechanisms of the MedDiet play a crucial role in vascular health, resulting in significant decreases in interleukin-6 (IL-6), high-sensitivity C-reactive protein (hs-CRP), and lipopolysaccharide (LPS) levels [14,32,33,34]. The underlying mechanisms that mediate these improvements are due to the diet’s rich content of monounsaturated fatty acids (MUFAs) and polyphenols—found mainly in EVOO—along with dietary fiber and antioxidants [35,36]. These components lead to improved mitochondrial function, a reduction in oxidative stress, and improvement in endothelial function, switching to healthier metabolic phenotypes [33,37], as well as modulating the expression of genes related to lipid metabolism and insulin sensitivity, among other mechanisms summarized by reviews highlighting in vitro and murine model approaches [2,38,39,40].

Addressing oxidative stress—a primary driver of brain aging and neurodegeneration—becomes critical for slowing dementia progression. The roles of oxidative stress and mitochondrial function are particularly significant under physiological stress, such as cerebral ischemia [41]. The MedDiet and its constituents, alongside vitamins C and E, reduce oxidative stress and consequently improve cognitive outcomes [42]. Mitochondrial dysfunction, as discussed by several preclinical models and subsequent reviews, has been linked to neuronal atrophy and AD [43,44,45]. Maintaining plasma membrane integrity and mitochondrial transmembrane potentials prevents the overproduction of reactive oxygen species (ROS), thereby reducing cellular malfunction and death [44]. Furthermore, Pollicino et al. [43] summarize MedDiet mechanisms, highlighting enhanced mitochondrial biogenesis, prevention of dysfunction and improved electron transport chain efficiency. The diet also appears to regulate processes such as autophagy and mitophagy in the liver, which are frequently dysregulated by a Western diet [45]. In particular, EVOO acts as a potent enhancer of mitochondrial health, increasing ATP generation, which results in improved cognitive scores in rat models [41].

#### 3.1.3. Stroke Prevention

The beneficial effects of the MedDiet on stroke prevention and recovery are significant. Adherence to the MedDiet results in reduced risk of total, ischemic, and hemorrhagic strokes [46,47,48]. The most reliable evidence was obtained from the PREDIMED trial [11], which demonstrated that adherence to the MedDiet results in lower stroke risk (HR = 0.58) when compared to the low-fat control group. This protective effect was corroborated by the CORDIOPREV trial [12], which obtained similar results, although the benefit was notably stronger in the male sex and in populations with significant comorbidities [47]. Chen et al. [48] reported more modest findings in the largest meta-analysis on the topic (n = 69,748), showing an overall reduction in total stroke relative risk (RR = 0.84), ischemic stroke risk (RR = 0.86), and hemorrhagic stroke risk (RR = 0.83). These differences between the meta-analysis and those from the RCTs could be attributed to a lack of control groups and less strict adherence in the observational studies included in the meta-analysis. However, the protective effect of the MedDiet in both Mediterranean and non-Mediterranean populations helps rule out other confounding variables related to geographic location or genetics [48]. A meta-analysis by Ungvari et al. [49] compared the results from 30 observational studies, finding a pooled HR = 0.88 in cohort studies, and a pooled HR = 0.54 in case–control studies. Despite these promising findings, it is essential to interpret the data from observational studies with caution. Unlike a controlled environment (e.g., PREDIMED), observational cohorts are susceptible to recall bias and “the healthy volunteer” bias, where participants adhering to a MedDiet tend to engage in other health-seeking behaviors that confound the results.

The protection against stroke is mainly due to the MedDiet’s favorable influence on CVRFs, as discussed in the above section, but also through direct vascular protective mechanisms. For example, the MedDiet has been shown to slow the progression of the IMT-CC—a marker of subclinical atherosclerosis—as demonstrated by the follow-up of two large RCTs [50,51]. Components of the MedDiet, in particular olive oil, have also been linked to improved functional recovery post-thrombectomy and arterial recanalization after ischemic stroke [52]. The MedDiet in patients with atrial fibrillation (Afib) treated with vitamin K antagonists was found to decrease the risk of transient ischemic attacks (TIA) and stroke (HR = 0.79) via a reduction in gut-derived lipopolysaccharide (LPS) [34,46]. The MedDiet’s ability to reduce inflammation and improve endothelial function, in conjunction with its modulation of pathways like the tryptophan–kynurenine pathway, contributes to its anti-atherosclerotic and antithrombotic effects, thereby reducing stroke risk [2,53]. Solid evidence, summarized in a recent systematic review by Bayes et al. [54], confirms the beneficial effects of the MedDiet on key cardiovascular risk factors in post-stroke adults, supporting its role in secondary stroke prevention.

#### 3.1.4. Metabolic Syndrome (MetS) and Type 2 Diabetes Mellitus (T2DM)

The MedDiet plays a significant role in the prevention and management of T2DM and MetS. Compliance and adherence to the MedDiet significantly reduce the risk of developing T2DM [55,56,57]. Specifically, participants with the highest adherence showed a low risk (HR = 0.48) for developing diabetes [55]. For patients already diagnosed with T2DM, the MedDiet has been shown to improve markers of metabolic and glycemic control, including fasting glucose, insulin levels, hemoglobin A1c (HbA1c), homeostatic model assessment of insulin resistance (HOMA-IR), and increased levels of HDL [58,59,60]. The MedDiet’s impact on body weight, BMI, and waist circumference directly influences the other components of the MetS [32,58]. Although based on smaller RCTs (n = 42), mild reductions in the main components of the MetS (BMI, blood pressure, glucose) were found, particularly when combined with isokinetic exercises. These smaller RCTs often lack the statistical power to reach significance for individual metabolic components, and their shorter durations may not capture the long-term effects or the magnitude of metabolic improvements observed in landmark clinical trials [61]. Comparatively, in larger trials (n = 3541), a 2-point improvement in the Mediterranean Diet Adherence Score (MEDAS) was associated with a 20% lower HR of developing T2DM [55]. A recent secondary analysis of the PREDIMED-Plus trial [62] yielded a lower incidence of T2DM with calorie-restricted MedDiet (AR = 9.5%) when compared with the regular MedDiet (AR = 12.0), suggesting that reduced calorie intake paired with the MedDiet could play an important role in T2DM control. Conversely, Chooi et al. [63] found no change in glycemic parameters despite having achieved weight loss in an RCT based on non-Mediterranean subjects, suggesting that the MedDiet effect in these populations could be lower than previously discussed.

The mechanisms involved include an increase in beneficial gut microbiota and subsequent short-chain fatty acid production, which enhances glycemic control and fat absorption [64,65,66]; however, these findings are primarily derived from murine models and secondary syntheses. The MedDiet also increases the release of incretin hormones like glucagon-like peptide-1 (GLP-1) and oxyntomodulin, which play an important role in glucose control [65,67]. Even in small-scale RCTs, the combination of the MedDiet and exercise significantly induced favorable changes in key inflammatory mediators, including cytokines such as resistin, adiponectin, and interleukin-10 (IL-10) [61,68]. The meta-analysis by Garza et al. [69] found that some of the specific components of the MedDiet, such as certain herbs and spices, directly improve glycemic parameters.

The MedDiet, particularly when combined with total alcohol abstinence, was found to effectively target organ-specific damage, particularly in the liver. Adherence is associated with reduced hepatic fat and improved enzyme levels, halting the progression of liver fibrosis. This offers a protective strategy against Metabolic Dysfunction-Associated Steatotic Liver Disease (MASLD) by restoring autophagy and mitophagy, two processes typically impaired by Western diets [70,71].

#### 3.1.5. Vascular Effects

Beneficial vascular effects appear to be the main mechanism of action explaining the protective role of the MedDiet against CVRFs, stroke, and T2DM/MetS. These vascular effects also appear to be protective in subjects with other forms of dementia. VaD is primarily caused by cerebrovascular disease, including stroke, chronic cerebral ischemia, and subcortical ischemic small vessel disease [5]. By reducing blood pressure—the most important population risk factor—as well as by improving lipid profiles and reducing inflammation, the MedDiet directly influences the main pathogenic factors of atherosclerosis, which play a fundamental role in the development of VaD [4]. The prevention of both ischemic and hemorrhagic stroke—as amply demonstrated in this review—is linked to consistent adherence to the MedDiet that would in turn reduce VaD incidence, given that stroke is a leading cause of VaD. Furthermore, the role of the MedDiet in managing T2DM and MetS is also critical for vascular health.

Insulin resistance, hyperglycemia and dyslipidemia, which are characteristic of these conditions, contribute directly to endothelial dysfunction, microvascular damage, and impaired cerebral blood flow [72]. By improving these metabolic parameters, the MedDiet helps preserve cerebrovascular integrity and function. Although evidence for a direct impact on hippocampal volume remains inconsistent, promising results from population-based longitudinal studies have shown that MedDiet adherence results in a decrease in the burden of subcortical ischemic small-vessel lesions, manifested by white matter hyperintensities, suggesting a direct cerebrovascular protective mechanism relevant to VaD [73]. This finding could be a direct effect of the increased circulating endothelial progenitor cells and the 50% reduction in carotid artery thickness among individuals adhering to the MedDiet, as reported by Maiorino et al. [51] in an RCT. Also relevant is the evidence of improved post-stroke recanalization in individuals who regularly consume the MedDiet and EVOO [52].

#### 3.1.6. Sleep and Mood Disturbances

Beyond its direct vascular and metabolic effects, the MedDiet also influences sleep and mood, providing a plausible mechanism for neuroprotection, as these factors are intimately linked to cognitive impairment and dementia risk [3,4]. Higher adherence to the MedDiet is associated with improved sleep quality, including reduced incidence of insomnia, more stable sleep architecture and longer duration of deep sleep [74,75,76]. Adherence to the MedDiet was shown to decrease BMI and improve positive airway pressure compliance in patients with obstructive sleep apnea (OSA), according to a small-scale RCT [77]. Regarding mood, the meta-analysis by Psaltopoulou et al. [78] found that adherence to the MedDiet reduced the risk of depression (RR = 0.68). In cross-sectional studies [79], greater adherence to the MedDiet correlates with a 28% reduction in the odds of developing symptoms of depression. The anti-inflammatory action of the MedDiet is primarily mediated by its influence on the NLR family pyrin domain containing 3 (NLRP3) inflammasome and Toll-like receptor 4 (TLR4) signaling pathways [66]. The MedDiet exerts positive effects on overall quality of life and mental health, especially in older adults who engage in physical activity, mainly through its anti-inflammatory and cardiovascular properties [80]. Furthermore, the varied nutrient influence on the gut microbiota, acting via the gut–brain axis, is thought to be another contributing mechanism, although supported only by preliminary data [81,82,83]. Most of the evidence suggests that the MedDiet is beneficial for sleep [84] and can reduce the likelihood and severity of depression, contrasting with less healthy dietary patterns [81,85]. Given the importance of the glymphatic circulation during deep sleep for cognitive function, the MedDiet, in combination with other lifestyle interventions (such as walking and physical activity) in patients with OSA resulted in improvement of inflammation and oxidative stress markers, suggesting a beneficial impact of the MedDiet on sleep apnea [86], closely linked with the progression of vascular cognitive impairment [87].

#### 3.1.7. Overall Effect and Dietary Recommendations

The attenuation of both vascular and metabolic risk factors by the MedDiet promotes a systemic health state, which may be optimal to preserve cognitive function and to reduce the incidence and severity of dementia. The MedDiet effectively addresses the main shared risk factors by reducing the atherosclerosis cascade and the microvascular damage that are both critical for dementia development. The high content of antioxidants and polyphenols in the MedDiet, combined with its capacity to decrease chronic inflammation, confers a neuroprotective effect in conjunction with direct metabolic and vascular protection. These systemic benefits provide basic mechanisms to mitigate the inflammation that drives both vascular and neurodegenerative pathologies, offering possible pathways for reducing the burden of all-cause dementias and potentially slowing disease progression. The MedDiet should be emphasized by clinicians in future dietary and non-pharmacological strategies specifically designed to mitigate the primary risk factors for dementia.

### 3.2. Effects of the MedDiet on Cognitive Decline, Alzheimer’s Disease and Vascular Dementia

There is ample support from the research articles reviewed here to conclude that the MedDiet provides a protective role against cognitive decline and neurodegenerative disorders. The main factors observed across multiple studies include the following: adherence to this dietary pattern is associated with better cognitive function, a reduced risk of MCI, and a lower incidence of AD and VaD. These protective effects, observed in various populations and different methodologies, appear again to be multifactorial, guided by a wide variety of bioactive compounds and physiological mechanisms.

These beneficial effects are supported by large meta-analyses with pooled data from 65,955 [88] and 12,458 participants [89], confirming the significant inverse association between MedDiet adherence and the risk of both all-cause dementia and AD. The populations included in these studies were older adults, and the MedDiet proved to be a protective factor in preventing cognitive decline. The meta-analysis by Singh et al. [90], focusing on prospective cohort studies, showed a notable reduction in the risk of developing both MCI (HR = 0.79) and AD (HR = 0.60). This reduction appears to be dose-dependent, with a risk reduction of 8% for every two-point increase in the Mediterranean Diet Adherence Screener (MEDAS) score, as proven by a meta-analysis focusing on MCI [89]. The most recent meta-analysis (2026) with over 730,000 participants [7] found a decreased risk of developing AD (RR = 0.92) and MCI (RR = 0.93) in high-adherence groups versus groups with low dietary adherence, and a 7% to 10% reduction in risk for every one-point increase in the MEDAS. These findings indicate a more modest risk reduction than previously estimated; however, the benefit per point of MEDAS adherence remains stable, suggesting that a high-adherence threshold may be difficult to reach in some populations. These data are supported by another recent meta-analysis by Fekete et al. [91], who found an overall decrease for both cognitive impairment (HR = 0.82) and dementia (HR = 0.89), with AD patients experiencing the largest risk reduction (HR = 0.70). Evidence remains robust even in non-Mediterranean populations. For example, Youn et al. [92] analyzed a cohort of 131,209 United Kingdom participants and found a decrease in dementia risk with higher adherence to the MEDAS (HR = 0.79) and to the Mediterranean-DASH Intervention for Neurodegenerative Delay (MIND) diet (HR = 0.73), demonstrating that these benefits persist away from the Mediterranean way of living.

In addition to the reduction in disease incidence, the MedDiet has demonstrated the capacity to directly improve individual cognitive scores when compared to a control diet [93]. Participants who supplemented their MedDiet with EVOO or mixed nuts [11,93] showed significant improvements in memory, frontal function and global cognition over a period of follow-up ≥ 4 years. In contrast, the control group experienced a de-cline in all cognitive scores, clearly demonstrating the direct neuroprotective role of the MedDiet and its components, such as EVOO or nuts [11,93]. Devranis et al. [94] found that most MedDiet interventions (n = 2609 participants), even of short duration, reported beneficial effects on cognitive function, both in healthy individuals and in subjects with MCI and AD, suggesting that the positive impact of the MedDiet occurs independently of the baseline cognitive status. This pattern is consistent with the findings analyzed above for metabolic risk factors; again, the combination of the MedDiet plus exercise (MedEx-UK) has demonstrated the most notable cognitive-protective effects in individuals at high risk of developing dementia [95].

The complex mechanisms responsible for the observed benefits are discussed in several reviews that primarily examine how the MedDiet functions in preclinical models [2,96,97]. The rich variety of antioxidants and anti-inflammatory compounds present in the MedDiet is regarded as the main causal agent. Long-chain omega-3 fatty acids found in fish and the abundant polyphenols of fruits, vegetables, cereals, and particularly in olive oil are emphasized. These compounds are thought to directly modulate pathways implicated in neurodegeneration. Although most of the evidence stems from preclinical studies, polyphenols from EVOO may influence tau hyperphosphorylation and amyloid-β (Aβ) aggregation, which are the two core pathophysiological mechanisms of AD [2,98]. Resveratrol, found in grapes and red wine, was found to also reduce Aβ aggregation, mitigate tau hyperphosphorylation, and promote neurogenesis through increased expression of neurotrophic factors like BDNF [99]. While in vitro results are promising, resveratrol exhibits poor bioavailability and rapid metabolic clearance [100]. Consequently, standard wine consumption does not achieve the systemic concentrations necessary to impact cognitive health or all-cause mortality [101]. Alcohol consumption is further discouraged by a trial by Mesquita et al. [102], who found statistically significant improvement in cognition only when the MEDAS was the inverse of wine consumption. Martínez-González [71] noted the lack of RCTs studying the effects of moderate alcohol consumption in the MedDiet; however, he reported clear evidence of alcohol toxicity in subcortical structures directly implicated in dementia progression. Furthermore, the World Health Organization (WHO) guidelines recommend total abstinence from alcohol in subjects with cognitive decline.

The main risk factors for dementia in the elderly, in particular AD and VaD, as well as the influence of the MedDiet, were analyzed in previous sections, in particular the strong link between cardiovascular and cerebrovascular health [103], and how the MedDiet may influence the progression of cognitive decline by reducing these risk factors. The components of the MedDiet, including unsaturated fatty acids and polyphenols, contribute to improving vascular endothelial function and mitigate cardiovascular risk factors such as dyslipidemia and hypertension [104,105]. Genetic predisposition appears to influence the outcomes, as high-risk APOE4 homozygous carriers appear to benefit more from high adherence to the MedDiet [106].

Adherence to the MedDiet appears to enhance cognitive outcomes through the modulation of neural dynamics, as evidenced by resting-state functional MRI (rs-fMRI). High adherence correlates with increased functional connectivity within fronto-parietal and temporo-occipital regions [107] and subcortical structures (thalamus, caudate, and putamen) [108]. These rs-fMRI findings translate clinically as improved executive function and attention [107], which are fundamental to the attenuation of symptoms in both AD and subcortical dementias. While some research shows no connectivity differences under rs-fMRI, MedDiet adherents often achieve superior cognitive performance despite lower connectivity, suggesting enhanced network efficiency [109]. At the cellular level, the diet may mitigate neurodegeneration by stabilizing the endocannabinoid system [110]. Murine models demonstrate that MedDiet polyphenols promote neurogenesis, as these compounds trigger neuronal differentiation and axonal-dendritic growth via specific molecular pathways [111].

A 2025 meta-analysis of observational studies by Wang et al. [8] provided structural evidence from MRI studies (n = 42,955), revealing a significant positive association between MedDiet adherence and brain volumes (total brain, gray matter, and hippocampus) and an inverse association with white matter hyperintensity [8,73], closely linked to reduced vascular cognitive impairment and VaD. An observational study by Cherian et al. [112] found that the MIND diet was more effective than its two separate components, the MedDiet and the DASH diet, in reducing post-stroke cognitive deterioration, suggesting that this dietary variation of the MedDiet might be more effective in the prevention of VaD than the MedDiet itself [113]. The observational phase of the DIETETICA trial [114] found that only 9.09% of patients with Cerebral Autosomal Dominant Arteriopathy with Subcortical Infarcts and Leukoencephalopathy (CADASIL) and cerebral amyloid angiopathy (CAA) had high MedDiet adherence, and none of the participants reached the polyphenol target. Both CADASIL and CAA are major genetic etiologies of cerebral small vessel disease, leading to increased risk of VaD and AD [115]. The second phase of the DIETETICA study, based on the implementation of MedDiet interventions, aims to clarify the relationship between these VaD precursors and strict MedDiet adherence [114].

The role played by the gut–brain axis has been addressed by emerging research about the beneficial effects of the MedDiet. It appears that the fiber, polyphenols, and healthy fats in the MedDiet can favorably alter the composition of the gut microbiota [82] by increasing the production of beneficial metabolites, such as short-chain fatty acids, which possess anti-inflammatory effects capable of mitigating systemic inflammation and oxidative stress that can compromise the integrity of the blood–brain barrier and accelerate AD pathology. In this regard, the consumption of yogurt and sour milk products fermented by probiotic bacteria appears to be beneficial to the gut–brain axis [2]. While the gut–brain axis offers a plausible explanation for how the MedDiet works, it remains largely a theoretical approach based on preclinical models. Definitive clinical measurements are needed to confirm these interactions in humans.

In addition to the solid evidence on the benefits of the MedDiet and EVOO consumption obtained from long-term longitudinal observational studies [116,117], there is a need for more RCTs conducted using EVOO as a pharmaceutical product [118,119]. Moreover, Jennings et al. [95] have demonstrated that interventions to improve MedDiet adherence, especially when combined with physical activity, could lead to improvements in cognitive function in older adults at risk of dementia. Furthermore, pilot studies [120] adding external components like coconut oil to the MedDiet in AD patients have shown preliminary improvements in global cognitive function, memory, and executive functions, providing a potential dietary alternative in places where the MedDiet is not readily available.

#### Overall Effects and Limitations

The cumulative evidence reviewed here, strongly supported by meta-analyses and large RCTs, positions the MedDiet as an optimal non-pharmacological strategy for cognitive decline and dementia. This protective effect appears to be multifactorial, functioning simultaneously through two major pathways: by mitigating systemic risk factors—such as hypertension, dyslipidemia, and insulin resistance—particularly related to VaD, and by providing direct neuroprotection through its rich profile of anti-inflammatory, antioxidant, and signaling-modulating compounds.

However, the interpretation of findings across the literature is complicated by significant heterogeneity in study designs. There is a lack of standardization in dietary definitions and adherence measures, such as the use of the MEDAS (based on absolute targets) [11] versus the MedDiet Score (based on median intake) [90]. Differences in the specific chemical composition of EVOO, particularly regarding polyphenol content, could significantly affect reported results, although this variability is difficult to measure and was not typically discussed across the included studies. Studied populations also varied widely; while research in Mediterranean populations (e.g., Italy, Spain) [11,12] has demonstrated significant outcomes, studies in non-Mediterranean regions (e.g., UK) [92] may potentially fail to replicate these effects due to cultural differences influencing adherence. Outcomes ranged from specific visuospatial assessments, such as Benton’s temporal orientation test or the clock drawing test [120], to broader clinical outcomes like the transition from MCI to AD [90]. Additionally, comparison with diverse dietary patterns, such as high-protein diets [13], makes direct comparisons between studies challenging and may contribute to the variable effects observed.

While preliminary structural evidence, such as improved brain volumes on MRI, and novel mechanisms like the gut–brain axis offer promising opportunities for future research, these findings must be interpreted alongside the multifactorial systemic benefits and the notable heterogeneity observed across different study designs. Despite these variations, the current body of data provides sufficient justification to promote Mediterranean Diet adherence as a viable, non-pharmacological approach for reducing the incidence and slowing the progression of all-cause dementia.

## 4. Mediterranean Diet in Comparison with Other Diets

Several RCTs and observational studies offer a comparison of the MedDiet with other alternative dietary interventions selected beyond the typical controls on regular diet. These comparisons highlight the most distinct advantages of the MedDiet. In some cases, the limitations of the MedDiet become apparent when it is compared against specialized diets designed to target specific health outcomes.

Low-fat diet: The MedDiet (enriched with EVOO or nuts) was proven to be superior to a low-fat diet for primary and secondary prevention of cardiovascular events by improving glycemic control, lipid profile and blood pressure [11,51,53]. For patients with established coronary heart disease, the MedDiet provided a significant reduction in major cardiovascular events (including MI, carotid stenosis and stroke); the beneficial effects were particularly pronounced in men when compared to the low-fat control diet [12,50].

Avocado-based MedDiet: The previously discussed efficacy is not absolute in all cohorts; one study in recent ischemic stroke/TIA patients found that while an avocado-based MedDiet achieved LDL-C reduction, it showed no significant differences in inflammatory markers, glucose, or stroke recurrence compared to a low-fat diet [121]. This specific finding is notable and could be attributed to the substitution of EVOO with avocado oil, potentially limiting the metabolic effects specifically associated with EVOO, suggesting that most cardioprotective benefits attributed to the MedDiet are highly dependent on the inclusion of EVOO as the main fat supply.

Ketogenic diets: In metabolic health, a Well-Formulated Ketogenic Diet (WFKD) was associated with a larger decrease in triglycerides compared to the MedDiet; however, the MedDiet led to a greater reduction in LDL cholesterol [122]. For weight loss in obese cohorts, the MedDiet showed more favorable body composition changes, offering greater preservation of fat-free mass and total body water compared to a very low-calorie ketogenic diet [15].

High-Protein Diet (HPD) and High-Fiber Diet (HFD): In women with prediabetes and obesity, an HPD demonstrated more pronounced metabolic benefits, including reduced insulin and better homeostasis model assessment of insulin resistance (HOMA-IR), along with improved glycemic variability when compared directly to the MedDiet, despite both diets leading to similar weight loss [123]. Conversely, for the management of T2DM, the MedDiet was found to enhance gastro-intestinal hormone release, specifically increasing glucagon-like peptide-1 (GLP-1) and oxyntomodulin release, as well as reducing post-prandial glucose when compared to the HFD vegetarian meal [67].

Dietary Approaches to Stop Hypertension (DASH): Both the MedDiet and DASH were proven to be effective in lowering blood pressure, with the MedDiet paired with salt restriction leading to the greatest reduction in office systolic blood pressure (−15.1 mmHg), while both the MedDiet and the DASH were equally effective in lowering 24 h ambulatory blood pressure [22]. However, when the MedDiet is not coupled with salt restriction, the resulting systolic blood pressure (−6.8 mmHg) was no longer superior to the systolic blood pressure (−11.4 mmHg) obtained from the DASH diet [124]. Regarding cognitive function, an RCT by Tangney et al. [125] found that the MedDiet was associated with slower global cognitive decline when compared to the DASH diet. While the DASH diet was less predictive of cognitive improvement, both diets positively correlated with improved cognition. Those subjects in the highest tertile of MedDiet adherence had an improvement in cognitive function of 0.034 standard units, as opposed to the highest tertile of DASH adherence with only 0.022 standard units of improvement when compared to the lowest adherence diets.

Mediterranean-DASH Intervention for Neurodegenerative Delay (MIND): The MIND diet, a Mediterranean-DASH hybrid, is based on plant-based foods. It places great emphasis on consuming large amounts of leafy green vegetables, nuts, and blueberries due to their potentially neuroprotective substances while limiting foods believed to be detrimental to brain health, such as cheese, red meat, and butter. In the context of neuroprotection post-stroke, the MIND diet demonstrated superior effectiveness in slowing cognitive decline compared to the traditional MedDiet and DASH diets, specifically showing benefits in semantic memory and perceptual speed [94,112]. An increase in potentially neuroprotective substances—such as folate, lutein, kaempferol, and β-carotene—probably explains the consistent finding of improved cognition observed with this hybrid diet [113,126]. Similarly to the regular MedDiet, the positive effects of the MIND diet are not limited to cognitive improvement. In a recent large prospective study (n = 384,512), Qin et al. [127] found a decreased risk of stroke (HR = 0.86) and heart failure (HR = 0.79), along with a reduced risk of non-atrial fibrillation arrhythmias. Despite the absence of large-scale clinical trials directly comparing the MedDiet and the MIND diet, observational prospective studies by Morris et al. [128] found that MIND adherence was a more powerful predictor of slower cognitive decline than the regular MedDiet, as follows: standardized β coefficients were 4.39 for the MIND diet, compared to 2.46 for the MedDiet and 2.60 for DASH. Hosking et al. [129] found that adherence to the MIND diet within an Australian cohort was associated with a 19% decrease in the odds of cognitive impairment, including a 53% reduction among higher adherence individuals. The regular MedDiet did not change the odds of cognitive impairment when adjusted for CVRFs. This discrepancy could probably be explained by the fact that the main effects of the MedDiet on cognition are exerted indirectly by mitigating the major CVRFs, or perhaps it might reflect the difficulties experienced by a non-native population in complying with the traditional Mediterranean diet.

It is important to consider that in most studies analyzed in this section, the MedDiet was the main intervention, while the other diets served as control groups (with exceptions such as the avocado-based MedDiet and MIND), which may introduce a risk of publication bias in favor of the MedDiet. Nevertheless, based on the available evidence, the MedDiet, especially when supplemented with EVOO, appears to be a robust dietary pattern for reducing vascular risk factors and slowing the progression of dementia. While some modified variants, such as the MIND diet or MedDiet combined with physical exercise, show potential for superior outcomes, these findings often stem from independent investigations of the MIND diet rather than direct, large-scale comparative RCTs.

On the whole, preliminary evidence suggests that the MIND diet may possess potent neuroprotective qualities, especially when combined with physical exercise and a modest caloric intake. However, these comparative advantages require further validation through controlled trials to determine if they outperform the traditional MedDiet in clinical settings. Given that the traditional MedDiet has been maintained for centuries, it will likely be easier for populations to sustain it over extended periods than the more restrictive MIND diet. Therefore, comparative studies on the long-term sustainability and clinical efficacy of both diets should be a priority for future research.

## 5. Extra-Virgin Olive Oil (EVOO)

The numerical quantification of the numerous beneficial effects of EVOO is challenging due to the scarcity of RCTs focusing solely on EVOO, separately from the other dietary components of the MedDiet. Methodological heterogeneity—as discussed in the Section: Overall Effects and Limitations—complicates this issue even further due to variations in the polyphenol content of the numerous varieties of EVOO and the significant differences in the patient cohorts studied. For these reasons, the primary evidence on the salutary effects of EVOO is currently based on preclinical mechanisms and the combined effects observed with the MedDiet.

### 5.1. Olive Oil and Risk Factors

#### 5.1.1. Stroke Prevention

In 2018, the PREDIMED trial [11], one of the most comprehensive and influential studies in the field, demonstrated that a MedDiet enriched with EVOO or nuts significantly reduced the incidence of cardiovascular problems compared to a low-fat diet. Stroke, the major risk factor for VaD, was the outcome with the largest reduction. Notably, a follow-up trial based on PREDIMED [53] found that an EVOO-enriched MedDiet reduced harmful metabolites of the tryptophan–kynurenine pathway, which have been mainly associated with an increased risk of atrial fibrillation, which in turn would increase the risk of stroke. These results showed that the anti-inflammatory action of EVOO involves cytokine signaling pathways, supporting the significant reduction of risk factors that may precipitate vascular cognitive decline. Nevertheless, these clinical trials fail to isolate the effects of EVOO and properly distinguish them from other MedDiet components.

Other studies have analyzed additional effects of the MedDiet on the outcome of stroke. Garcia-Cabo and colleagues [52] conducted an observational study on 239 ischemic stroke patients and demonstrated that olive oil consumption by itself was associated with improved recovery and higher rates of complete arterial recanalization, unlike the overall MedDiet adherence, which did not consistently predict outcomes after thrombectomy. This may confirm that EVOO produces unique vascular effects separate from those obtained with the MedDiet alone.

#### 5.1.2. Metabolic Effects

EVOO is rich in MUFAs, predominantly oleic acid, and polyphenols such as hydroxytyrosol (HT) and oleocanthal (OLC). These compounds reduce oxidative stress, enhance endothelial function, and modulate inflammatory cascades implicated in both atherosclerosis and cerebral small-vessel disease, all intimately related to the pathogenesis of vascular dementia. As reviewed by several authors [2,39], EVOO reduces the level of inflammatory markers (CRP, IL-6, TNF-α), improves lipid profiles (raising HDL-C and lowering LDL-C) and enhances insulin sensitivity, all of which match the primary modifiable risk factors described by Ng et al. [4]. A 2025 meta-analysis by Du et al. [130] found a 22% decreased risk of T2DM in RCTs compared with 13% in cohort studies; the authors determined that the ideal dose of EVOO ranged between 10 and 20 g/day. Focusing on these specific molecular mechanisms, a review [131] suggests that improved blood sugar control in EVOO consumers may be explained by multiple mechanisms, including the stimulation of the PI3K/Akt pathway, IRS-1/2 phosphorylation, and the promotion of GLUT4 translocation, which therefore reduce insulin resistance. However, since these findings are primarily based on in vitro models, it remains difficult to determine how much these pathways contribute to the clinical effects seen in humans.

Studies evaluating the effects of the MedDiet in subjects with MetS and T2DM [26,35,39] have consistently reported that EVOO contributes to reductions in waist circumference, triglycerides, and fasting glucose, while also improving HDL and reducing systemic inflammatory factors directly implicated in cerebrovascular risk. In particular, a recent cohort study [132] demonstrated that sporadic and less frequent EVOO consumption was associated with increased odds of developing obesity (OR = 5.1) regardless of the remainder of the diet. The polyphenols present in EVOO appear to modulate enzymatic routes such as endothelial nitrogen oxide (NO) synthase and the response to NO, reducing reactive oxygen species (ROS), suppressing endothelin-1 (ET-1) and enhancing calcium signaling, among other mechanisms, which altogether help improve cerebral perfusion and microvascular health [133]. Polyphenols have also been shown to activate the Nrf2 pathway, upregulating superoxide dismutase (SOD) and glutathione-S-transferase [134], and promoting mitochondrial biogenesis and respiratory efficiency [135], mechanisms that contribute to ROS neutralization, thereby reducing inflammation. The MUFAs, like oleic acid, act as lipid sensors in the gut, promoting satiety and consequently reducing obesity [132].

Overall, multiple reviews have confirmed the combined effects of EVOO with the MedDiet, but the individual role of EVOO consumption has rarely been studied separately. Nevertheless, EVOO appears to be the most potent dietary component. Research isolating the role of concentrated MUFAs and polyphenols found in EVOO, as well as vitamin E and carotenoids, has proven to deliver an antioxidant effect that is directly linked to the specific reduction in carotid atherosclerosis (IMT-CC), mitigating vascular risk factors, MetS, depression and stroke through reduced inflammation, all of which constitute major risk factors for dementia development [28,31].

#### 5.1.3. Cardiovascular Effects

A focused search on the effects of EVOO on the main CVRFs showed that EVOO decreases markers such as serum thromboxane B2 [136], as well as circulating levels of C-reactive protein (CRP) and IL-6 [137], as validated by specific RCTs and subsequent meta-analyses that isolated EVOO as the primary intervention. Specific components, notably HT and oleuropein (OLE) aglycone, exert these benefits in cellular models by reducing adhesion molecules and inhibiting enzymes like COX-1/2 [138,139]. Beyond inflammation, solid findings have shown improvement of serum lipids, with single intakes of olive oil and tomatoes decreasing total plasma cholesterol (−7.46 mg/dL), triglycerides (12.57 mg/dL) and increasing plasma HDL (+2.39 mg/dL) levels [140]. The inverse correlation of urinary polyphenols and CVRF incidence objectively positions EVOO as the frontline beneficial factor of the MedDiet [27]. In particular, HT (from OLE aglycone hydrolysis) was found to be the factor with the best correlation to decrease the incidence of CVRFs, mainly through decreased LDL oxidation [27]. In contrast, broader reviews found no effects of EVOO on lipoprotein metabolism [141], while a more updated review reported that EVOO lowered LDL and increased HDL [142]. This discrepancy could be explained by the modest improvements in the lipid profile found in previous RCTs [140], given the variability in EVOO selection (in particular for polyphenol and MUFA content) and the generally less strict adherence found in the observational studies compared to RCTs. Table 1 summarizes the effects and proposed underlying mechanisms of EVOO polyphenols and other Mediterranean diet constituents.

Other studies have indicated that high polyphenol EVOO can lead to a decrease in both peripheral and central systolic blood pressure (−2.5 and −2.7 mmHg, respectively) [143,144], but no significant differences in diastolic blood pressure [144], suggesting that the antihypertensive impact of EVOO is modest in comparison to its other cardiovascular benefits. A diet rich in MUFA appeared to prevent the postprandial deterioration of endothelial function, as proven by two RCTs [140,145], increasing vasodilation and reducing markers like vascular cell adhesion molecule-1 (VCAM-1) [145], intercellular adhesion molecule-1 (ICAM-1) or leukocyte function-associated antigen-1 (LFA1) [140]. These molecular changes likely serve as a possible explanation for the observed vasodilatation following olive oil consumption [137]. The anti-atherogenic properties and vasodilatory capacity of EVOO, secondary to increased NO bioavailability, have previously been emphasized by several reviews [142,146]. A recent systematic review confirmed the pivotal role of EVOO in the reduction in CVRFs and stroke attained at high daily doses (20–30 g/day) [144].

### 5.2. Effects of Olive Oil on Cognitive Decline

EVOO is a fundamental nutrient of the MedDiet and the MIND diet with potential neuroprotective properties. Higher adherence to these dietary patterns results in slower cognitive decline and a reduced incidence of AD, especially in older adults [113]. The protective effect of EVOO may be largely attributed to the high content of MUFAs, along with a wide range of bioactive phenolic compounds, among other essential nutrients.

Several potential mechanisms of neuroprotection result from consumption of EVOO. Its phenolic compounds, such as OLC, HT, OLE aglycone, and luteolin, appear to be critical for its beneficial effects [147,148,149]. These compounds have been linked in preclinical studies to direct modulation of key mechanisms of AD, including the effects of OLE and OLC in inhibiting the assembly of amyloid-β protein into neurotoxic oligomers and fibrils and facilitating the disaggregation of already preformed amyloid structures [1,150,151,152]. Similarly, EVOO polyphenols contribute to reducing tau phosphorylation and aggregation, as discussed in several reviews examining preclinical models [1,147,148].

Apart from direct amyloid and tau modulation, in vitro studies have shown that EVOO exerts significant anti-inflammatory and antioxidant effects. Specifically, phenolic compounds can reduce chronic neuroinflammation by decreasing microglial activation and lowering levels of inflammatory cytokines [148,149,150]. They also inhibit pathways like p38 MAPK (mitogen-activated protein kinase), involved in oxidative stress and neurodegeneration, contributing to breaking the amyloid toxicity cycle and reducing neural death [153]. In addition, EVOO has been shown to preserve the integrity of the blood–brain barrier, improving synaptic function [147]. The activation of autophagic processes, mainly through the AMPK/mTOR pathway and sirtuin-mediated gene expression by compounds like OLE, helps to clear cellular debris and to improve mitochondrial function, offering protection against AD [150,154].

The translation of preclinical evidence into clinical outcomes is supported by RCTs and observational data showing that EVOO consumption provides clear cognitive benefits. In particular, daily consumption of EVOO in individuals with MCI has been shown to significantly improve memory scores, reduce blood–brain barrier permeability, and enhance resting-state brain connectivity, particularly involving the hippocampus [155,156]. This was accompanied by a decrease in plasma Aβ42/Aβ40 and p-tau/t-tau ratio, suggesting a direct modulation of AD pathology [156].

Therefore, EVOO could exert a neuroprotective effect against AD mainly through its polyphenols and their interaction with tau and Aβ amyloid pathways, as supported by preclinical evidence [157,158]. Regarding each polyphenol effect, OLE and HT stabilize Aβ monomers and redirect the process towards the formation of non-toxic aggregates, thereby inhibiting the formation of toxic oligomers [157]. OLE and HT also contribute to other pathways by inhibiting the fibrillization of tau protein, decreasing the formation of neurofibrillary tangles [158]. OLE and verbascoside contribute to the maintenance of synaptic integrity by upregulating synaptic proteins (SNAP-25, PSD-95) and by modulating calcium signaling [158]. OLC enhances the cerebral clearance of Aβ amyloid by improving its efflux across the blood–brain barrier and activating the autophagy pathway (AMPK/ULK1), contributing to the removal of existing amyloid deposits [157]. While less studied, ligstroside, present in olive cultivars, has been shown to improve spatial working memory [158]. Collectively, the polyphenols present in EVOO contribute to the significant reduction of amyloid deposits in the hippocampus and cerebral microvasculature [157,158]. Although the clinical potential of polyphenols is significant, these effects have been primarily demonstrated in vitro and in murine models [147,150,154,157,158]. Future research must determine how these mechanisms translate into clinical practice by isolating EVOO and its polyphenols.

Stavrinou et al. [159] used olive oil as a placebo in an RCT and found only minor cognitive effects that were less significant than those in the active control group (pure antioxidant supplementation). While this suggests that purified EVOO components may be more efficacious than whole EVOO, the nutritional implementation of a supplement on a population basis would be significantly more challenging than promoting the consumption of EVOO. Further support for the positive effects of EVOO is provided by a systematic review of 88 studies conducted by Christodoulou et al. [160], concluding that EVOO is the most critical component of the MedDiet for reducing the risk of dementia. This analysis showed that an EVOO-rich MedDiet leads both to improved cognitive function and to a reduced incidence of MCI when compared with a control diet. Similarly, after a 6.5-year follow-up, the MedDiet supplemented with EVOO was associated with reduced odds of developing MCI (OR = 0.34) and improved cognition when compared with a low-fat diet [161]. EVOO given in the context of the MedDiet elicited better cognitive scores when compared with other vegetable oils used within the same diet; these results were significant even over the short follow-up period of 12 months [162]. EVOO supplementation was also proven to be superior to the MedDiet supplemented with nuts [161]. Observational studies in MCI patients have shown that EVOO consumption enhances general cognitive performance, especially in global cognition and verbal fluency, as well as maintaining stable function, even in high-risk APOE4 carriers [163].

Therefore, EVOO is amply supported as the most potent component of the MedDiet for cerebrovascular protection. The scarcity of independent RCTs makes quantifying EVOO’s individual numerical effects challenging. Nevertheless, the positive effects of EVOO, both independently and synergistically with the rest of the Med-Diet components, are associated with unique health benefits, including improved post-stroke arterial recanalization and mitigation of key vascular risk factors. The mechanisms for this efficacy are driven by high concentrations of MUFAs and polyphenols that reduce inflammation and enhance endothelial function. Collectively, there is a robust body of clinical evidence on stroke reduction and metabolic benefits that confirms EVOO’s role as an essential, non-pharmacological intervention against VaD and AD.

## 6. Conclusions

In summary, there is substantial evidence supporting the benefits of EVOO and the MedDiet for the control of vascular risk factors, including improvement of hypertension by reducing systolic and diastolic blood pressure; dyslipidemia by lowering triglycerides and LDL cholesterol and increasing HDL cholesterol; diabetes by improving fasting glucose, insulin levels, and HbA1c; and lowering BMI, central adiposity and obesity, potentially leading to the prevention of cardiovascular disease and stroke. The mitigation of vascular risk factors may contribute to the clinical improvement of MCI, AD and VaD. The mechanisms involved in these positive effects are mediated mainly by control of inflammation, demonstrated by the significant decrease in inflammatory markers and by the improvement of mitochondrial function, reduction in oxidative stress and overall optimization of endothelial and vascular physiology contributing to reduce atherosclerosis and thrombosis, thereby lowering stroke risk. The MedDiet also exerts significant influence on sleep architecture, including improvements in quality and duration that are manifested as a reduction in sleep apnea, insomnia and depressive symptoms. The MedDiet has also demonstrated beneficial effects on the gut–brain axis mediated by probiotic bacteria, and it has been shown to protect patients with MASLD and hepatic steatosis, particularly when the MedDiet is followed without alcohol consumption from wine. In addition to the positive vascular actions of EVOO, a direct effect on the pathophysiology of neurodegenerative conditions is suggested by the demonstrated effect of polyphenols on tau hyperphosphorylation and amyloid aggregation in AD models.

The positive effects of the MedDiet and EVOO on cognition in the elderly, MCI, AD, and VaD, are well supported by the extensive observational, epidemiological and clinical studies reported here. Despite the comprehensive CORDIOPREV and PREDIMED studies, the main limitation of the literature in this field is the lack of placebo-controlled RCTs studying EVOO as a pharmaceutical product. Variations in participant demographics, the lack of standardized adherence measures, and the differing polyphenol profiles of the olive oils used suggest that the MedDiet may not produce uniform cognitive outcomes across all global populations. Specifically, there is a significant need for large-scale longitudinal trials designed to evaluate the potential of EVOO intervention to favorably influence the clinical course of MCI, AD, and VaD. Future trials should contribute to consolidate the role of EVOO and the MedDiet as effective preventive and supportive nutritional strategies for dementias in the elderly.

## Figures and Tables

**Table 1:** Mechanisms of action of the bioactive components of the Mediterranean Diet.

Component	Bioactive Compounds	Mechanism/Risk Factor Addressed
Whole grains, legumes, vegetables	Fiber	Obesity/MetS/T2DM (BMI, waist
		circumference), gut microbiota
		health (via SCFA production)
Fish	Long-chain Omega-3s	Inflammation
		Lipid profile
Extra-virgin olive oil	MUFAs	Dyslipidemia, hypertension,
	Polyphenols	inflammation, endothelial dysfunction, stroke
	MUFA: Oleic Acid (Omega-9 fatty acid)	Endothelial function, vasodilation, VCAM-1 modulation, blood–brain barrier permeability
	Polyphenols: Hydroxytyrosol, Oleocanthal, Oleuropein aglycone, Ligustroside, Verbascoside	Inflammation (IL-6, hs-CRP), oxidative stress, amyloid/tau modulation, inflammatory pathways (p38 MAPK, mTOR, COX-1/2, ET-1), mitochondrial function

## Data Availability

All data is contained within the article.

## Abbreviations

The following abbreviations are used in this manuscript:

AD	Alzheimer’s disease
Afib	Atrial fibrillation
AMPK/mTOR	Adenosine monophosphate-activated protein kinase/mammalian target of rapamycin
AMPK/ULK1	AMPK/Unc-51 like autophagy activating kinase 1
APOE4	Apolipoprotein E4
AR	Absolute risk
Aβ	Amyloid-β
Aβ42/Aβ40	Amyloid-beta 42 to 40 ratio
BDNF	Brain-derived neurotrophic factor
BMI	Body mass index
CAA	Cerebral amyloid angiopathy
CADASIL	Cerebral Autosomal Dominant Arteriopathy with Subcortical Infarcts and Leukoencephalopathy
CKD	Chronic kidney disease
CORDIOPREV	CORonary Diet Intervention with Olive Oil and Cardiovascular PREVention
COX-1/2	Cyclooxygenase-1 and 2
CRP	C-reactive protein
CVRFs	Cardiovascular risk factors
DAI	Composite Dietary Antioxidant Index
DASH	Dietary Approaches to Stop Hypertension
EMBASE	Excerpta Medica DataBASE
EVOO	Extra-virgin olive oil
GLP-1	Glucagon-like peptide-1
HbA1c	Hemoglobin A1c
HDL	High-density lipoprotein
HFD	High-Fiber Diet
HOMA-IR	Homeostatic model assessment of insulin resistance
HPD	High-Protein Diet
hs-CRP	High-sensitivity C-reactive protein
HT	Hydroxytyrosol
ICAM-1	Intercellular adhesion molecule-1
IL	Interleukin
IMT-CC	Intima-Media Thickness of the Common Carotid
LDL	Low-density lipoprotein
LFA1	Leukocyte function associated antigen-1
LPS	Lipopolysaccharide
MCI	Mild cognitive impairment
MEDAS	Mediterranean Diet Adherence Score
MedDiet	Mediterranean diet
MetS	Metabolic syndrome
MI	Myocardial infarction
MIND	Mediterranean-DASH Intervention for Neurodegenerative Delay
MRI	Magnetic resonance imaging
MUFAs	Monounsaturated fatty acids
NLRP3	NOD-, LRR- and pyrin domain-containing protein 3
NO	Nitric oxide
OLC	Oleocanthal
OLE	Oleuropein aglycone
OSA	Obstructive sleep apnea
p-tau/t-tau	Phosphorylated tau to total tau ratio
p38 MAPK	p38 mitogen-activated protein kinase
PREDIMED	PREvención con DIeta MEDiterránea
PSD-95	Postsynaptic density protein 95
RCTs	Randomized controlled clinical trials
rs-fMRI	Resting-state functional MRI
sd-LDL-C	Small dense low-density lipoprotein cholesterol
SNAP-25	Synaptosomal-Associated Protein 25
T2DM	Type 2 diabetes mellitus
TIA	Transient ischemic attack
TLR4	Toll-like receptor 4
VaD	Vascular dementia
VCAM-1	Vascular cell adhesion molecule-1
VCI	Vascular cognitive impairment
VCID	Vascular Contributions to Cognitive Impairment and Dementia
WFKD	Well-formulated ketogenic diet
WHO	World Health Organization
